# Structural and Magnetic Properties of Carbon-Based Nanocomposites Containing Iron Oxides: Effect of Thermal Treatment Atmosphere

**DOI:** 10.3390/nano15161241

**Published:** 2025-08-13

**Authors:** Daniel Hidalgo-Montoya, Mario A. Millán-Franco, John Betancourt, Lorena Marín, Luis A. Rodríguez, Jesús A. Tabares, Milton Manotas-Albor, César Magén, Manuel N. Chaur, Edgar Mosquera-Vargas, Renso Visbal, Malka Mora

**Affiliations:** 1 Departamento de Química, Facultad de Ciencias Naturales y Exactas, Universidad del Valle, Cali AA 25360, Colombia; hidalgo.daniel@correounivalle.edu.co (D.H.-M.); manuel.chaur@correounivalle.edu.co (M.N.C.); 2 Instituto de Física, Universidad Nacional Autónoma de México, Circuito de Investigación Científica s/n, Ciudad Universitaria, A.P. 20-364, Coyoacán 04510, Mexico; mario.millan@correounivalle.edu.co; 3 Centro de Excelencia en Nuevos Materiales (CENM), Universidad del Valle, Cali AA 25360, Colombia; betancourt.john@correounivalle.edu.co (J.B.); marin.lorena@correounivalle.edu.co (L.M.); luis.a.rodriguez@correounivalle.edu.co (L.A.R.); edgar.mosquera@correounivalle.edu.co (E.M.-V.); 4 Grupo de Transiciones de Fase y Materiales Funcionales, Universidad del Valle, Cali AA 25360, Colombia; 5 Grupo de Películas Delgadas, Universidad del Valle, Cali AA 25360, Colombia; 6 Servicio Geológico Colombiano, Dirección de Laboratorios, Cali 760031, Colombia; 7 Grupo de Metalúrgica Física y Teoría de Transiciones de Fase (GMTF), Universidad del Valle, Cali AA 25360, Colombia; jesus.tabares@correounivalle.edu.co; 8 Grupo de Investigación en Física Aplicada (GIFA), Departamento de Física y Geociencias, Universidad del Norte, Barranquilla 080006, Colombia; manotasm@uninorte.edu.co; 9 Instituto de Nanociencia y Materiales de Aragón (INMA), CSIC-Universidad de Zaragoza, 50009 Zaragoza, Spain; cmagend@unizar.es

**Keywords:** carbon-based nanocomposites, iron oxide nanoparticles, hematite, wet impregnation, thermal treatment atmosphere, weak ferromagnetic

## Abstract

Carbon-based nanocomposites coated with iron oxides were synthesized using a wet impregnation method with thermally annealed coal and an iron nitrate precursor. The influence of the thermal treatment atmosphere (air, vacuum, or nitrogen) on the morphology, structure, and magnetic properties of the nanocomposites was examined by X-ray diffraction, Raman spectroscopy, and transmission electron microscopy. It was found that the vacuum thermal treatment produced carbon-based nanocomposite containing iron oxide with the highest crystallinity, according to XRD analysis, while also inducing the greatest degree of structural defects in the carbon matrix, as evidenced by Raman analysis. Mössbauer spectroscopy confirmed that all thermal treatment methods promote the formation of the hematite phase, which was found to be the only phase formed in the air-treated nanocomposites, whereas traces of magnetite and the formation of Fe(OH)_3_ were detected in the vacuum- and nitrogen-treated nanocomposites, respectively. Magnetic characterization revealed that all nanocomposites exhibit ferromagnetic-like behavior, attributed to the weak ferromagnetic nature of hematite. The best magnetic response (highest saturation magnetization with the widest hysteresis loop) was observed in the vacuum-treated nanocomposites. These findings collectively demonstrate that the synthesis atmosphere plays a crucial role in tailoring the structural and magnetic characteristics of carbon-based iron oxide nanocomposites, offering pathways for their optimization in applications such as catalysis, environmental remediation, or sensing technologies.

## 1. Introduction

Coal remains one of the most abundant and widely distributed natural resources worldwide, with substantial reserves of anthracite and bituminous coal across multiple regions. Historically, its primary use has been as a fuel source. However, the ongoing global energy transition is transforming its role, prompting a shift toward the development of high-value materials derived from coal that meet current technological and market demands. This shift reflects the need to reduce environmental impact while maximizing the functional potential of coal in advanced applications [1,2,3]. Carbonaceous materials exhibit remarkable versatility due to their tunable chemical structure, surface area, and porosity. Their inherent chemical and thermal stability, non-toxic nature, natural abundance, and low production cost make them ideal candidates for a wide range of technological and industrial applications such as hydrogen storage, water treatment, and electrochemial CO_2_ reduction, etc. [4,5,6,7,8,9]. These properties can be further enhanced by incorporating into their structure uniformly distributed metal particles [10]. In particular, the integration of magnetic nanoparticles, such as iron oxides, has drawn significant attention due to their synergistic interactions with carbon [11,12,13].

Among the different iron oxide phases, hematite (α-Fe_2_O_3_) stands out due to its exceptional chemical stability, non-toxicity, and widespread availability, making it a highly promising material for various technological applications [14,15,16,17]. Its high corrosion resistance and thermodynamic stability allow for use in extreme environments, while its semiconducting nature and visible-light absorption capabilities make it valuable for photocatalysis and energy conversion [13,18]. Additionally, at the nanoscale, hematite nanoparticles exhibit unique magnetic properties, including weak ferromagnetism and high coercivity, which enhance its suitability for applications in magnetic storage and biomedical technologies. When incorporated into carbon-based composites, hematite further improves electrochemical performance, catalytic efficiency, and structural durability, making it an essential component in energy storage devices [13] and environmental remediation [19,20].

Understanding how these materials evolve under different atmospheric conditions is crucial for optimizing their structural and magnetic properties [21,22,23]. The thermal treatment atmosphere significantly influences the crystallinity, phase composition, and morphology of the nanocomposites, directly impacting their functional performance [24,25]. Variations in oxygen availability, temperature, and reactive gas composition affect the oxidation state of iron, the formation of carbon structures, and the interfacial interactions between iron oxides and carbon. Investigating these effects provides valuable insights for tailoring synthesis conditions, ultimately enhancing the performance and applicability of these materials in cutting-edge technologies [26,27,28]. The structural and morphological characteristics of iron oxide nanomaterials are highly sensitive to the atmosphere employed during thermal treatment. In a study by Slimani et al. (2021), hematite nanoparticles were synthesized under air, nitrogen, and argon atmospheres via a sol–gel route followed by thermal annealing at 500 °C. The results demonstrated that the crystallite size, phase purity, and morphology were significantly influenced by the annealing environment. Specifically, thermal treatment in nitrogen yielded smaller crystallite sizes and reduced crystallinity compared to air, indicating that inert atmospheres hinder grain growth and affect phase formation pathways. The authors also observed variations in surface area and magnetic behavior, suggesting a direct correlation between the synthesis atmosphere and the functional properties of the nanomaterials [29]. Similarly, Kundu et al. (2024) investigated the thermal decomposition of ferrocene under different atmospheres (air, O_2_, and N_2_), revealing that distinct iron-containing phases could be selectively obtained by simply adjusting the gaseous environment. Under air and O_2_, pure hematite (α-Fe_2_O_3_) was formed, whereas N_2_ conditions favored the formation of mixed phases including Fe_3_C (cementite) and α-Fe_2_O_3_, attributed to limited oxidation and carbon incorporation. These findings confirm that the thermal atmosphere plays a pivotal role not only in phase composition but also in particle morphology, crystallite size, and surface characteristics [30]. In another related study, Alcalá and Real (2006) showed that oxidizing atmospheres such as air promote the formation of hematite (α-Fe_2_O_3_), while reducing atmospheres (e.g., N_2_/H_2_) stabilize phases like magnetite (Fe_3_O_4_) and even metallic iron (α-Fe), with the silica matrix acting as a diffusion barrier that favors metastable phase retention and limits crystal growth [31]. Complementarily, da Cunha et al. (2021) observed that sequential calcination in air followed by hydrogen reduction transformed oxide nanoparticles into their metallic counterparts (Co^0^ and Fe^0^), significantly altering dispersion, crystallinity, and support interactions, factors that directly impacted their catalytic performance and morphological uniformity [32]. More recently, Vale et al. (2025) reported a dual-atmosphere thermal approach in which Fe^3+^ impregnated into MCM-48 mesoporous silica was first calcined in air to form hematite, and then annealed in argon at 800 °C to generate a hematite–magnetite mixture. This stepwise treatment not only preserved the spherical morphology and mesoporous order but also enhanced photoacoustic signal performance due to improved thermal confinement and FeNPs distribution [33]. Collectively, these studies highlight that carefully controlled thermal environments are essential to tune nanoparticle size, phase stability, and functional properties of metal–silica nanocomposites for applications ranging from catalysis to biomedical imaging. In this work, we have synthesized iron oxides-coated carbon nanocomposites by a simple wet impregnation method. A comprehensive characterization by scanning electron microscopy (SEM), transmission electron microscopy (TEM), X-ray diffraction (XRD), Raman and Mössbauer spectroscopy, and magnetometry has allowed us to investigate the influence of the synthesis atmosphere (air, vacuum, or N_2_) on the structural and magnetic properties of these heterostructures.

## 2. Materials and Methods

### 2.1. Thermal Annealing of Amorphous Mineral Coal in an Inert Atmosphere

The mineral coal used in this study was sourced from the Fortuna mine (Departamento de Santander, Colombia), and classified as semi-anthracite according to ASTM D388 [34]. The proximate analysis of the coal was carried out in accordance with ASTM methods D3174 for ash content (8.83%) [35], D3172 for fixed carbon (80.16%) [36], D3175 for volatile matter (10.41%) [37], D5865 for calorific value (14,033 BTU/lb), and D4239 for sulfur content (0.69%) [38,39]. An ultimate analysis to determine the nitrogen (1.48%), hydrogen (3.15%), and carbon (78.45%) content was also conducted based on ASTM D5373 [40]. Subsequently, 8 g of the coal sample was placed in a rectangular magnesium oxide crucible and placed inside a Nabertherm LHT 02/18 high-temperature furnace. The sample was heated to 1700 °C at a rate of 10 °C/min under a constant argon flow at 150 L/h and then held at this temperature for 2 h. After the heating process, the sample was allowed to cool inside the furnace and was retrieved once it reached room temperature.

### 2.2. Synthesis of Carbon-Based Nanocomposites Coated with Iron Oxides

**Figure 1:**
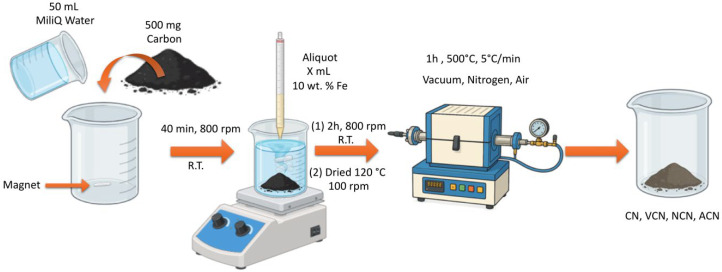
Schematic representation of the synthesis procedure for Fe_x_O_y_/C nanocomposites.

Iron deposition was carried out using a wet impregnation method. A quantity of 500 mg of the annealed mineral coal (AMC) and 50 mL of water obtained by Milli-Q system were placed in a 300 mL beaker at room temperature. The mixture was stirred at 800 rpm for 40 min. Subsequently, 11 mL of a 0.09 M Fe(NO_3_)_3_∙9H_2_O solution was added to the carbon suspension, resulting in an iron loading of 10 wt%. The mixture was stirred at 800 rpm for an additional 2 h at room temperature. Afterward, the suspension was dried in air at 70–80 °C using a heating plate equipped with a thermocouple, and the resulting material was divided into three equal portions. One portion was subjected to thermal treatment at 500 °C for 1 h, with a heating rate of 5.0 °C/min, in a tubular furnace (MTI Corporation GSL1500X) in air. The other two portions were treated under the same thermal conditions but in different environments: one in reduced air pressure (~0.09 bar), initially achieved using a mechanical pump and subsequently maintained as a static vacuum after sealing the system and the other in a dynamic N_2_ atmosphere (0.5 mL/min). The resulting materials were labeled as ACN 10, VCN 10, and NCN 10, where A, V, and N denote the atmospheres used during synthesis. The sample without iron was labeled CN. A schematic representation of the synthesis process is shown in Figure 1.

### 2.3. Sample Characterization

Structural and phase analysis of the samples was performed by X-ray diffraction (XRD) using a Bruker D8 Advance Eco diffractometer with CuK_α_ radiation (λ = 0.15418 nm, 40 kV, 25 mA). Data were collected at room temperature and over a 2θ angle range of 10–80°, with a scan rate of 0.01°. Crystalline phase detection was carried out by comparing the obtained patterns with reference data from the Inorganic Crystallography Open Database (COD), using the X’pert HighScore Plus software (Malvern Panalytical B.V., Almelo, The Netherlands)). Local morphological, crystalline and compositional analysis were performed by scanning electron microscopy (SEM) using a Phenom Pro X microscope, equipped with an energy-dispersive X-ray spectroscopy (EDS) detector from Oxford Instruments, and by high-resolution transmission electron microscopy (HRTEM) in a Thermo Fisher Scientific image-corrected Titan 60–300 microscope operating at 300 kV. For these experiments, the samples were ultrasonically dispersed in isopropyl alcohol and drop-cast onto an ultrathin holey carbon-coated copper grid. A complementary analysis of the type of iron oxides presented in the samples was performed through Raman and Mössbauer experiments. Raman spectra were recorded using a Jasco RMS 4500 Raman spectrometer, employing a 532 nm (2.34 eV) laser with a power below 2 mW to prevent overheating. Mössbauer spectra were acquired at room temperature using a constant-acceleration spectrometer with a ^57^Co/Rh radioactive source (~5 mCi). The spectra were fitted using the MOSFIT program [41] and the isomer shift (IS) was referenced to α-Fe. Finally, magnetic characterization was performed by measuring the hysteresis loops of the specimens using the vibrating sample magnetometer (VSM) module of a Physical Properties Measurement System (PPMS) from Quantum Design^®^. The metal content of the samples was determined using an Agilent 5110 VDV ICP-OES instrument. The measurements were carried out in axial view mode with four replicates per sample. The integration time was set to 6 s. A Seaspray nebulizer coupled with a double-pass cyclonic spray chamber was used for sample introduction. The operating conditions included an RF power of 1200 W, a peristaltic pump speed of 12 rpm, and the following gas flow rates: plasma gas at 12 L/min, nebulizer gas at 0.70 L/min, and auxiliary gas at standard instrument settings. Calibration curves were obtained using certified standard solutions, and all measurements were performed under optimized and stabilized plasma conditions to ensure accuracy and reproducibility.

## 3. Results and Discussion

### 3.1. Structural Analysis from X-Ray Diffraction

**Figure 2:**
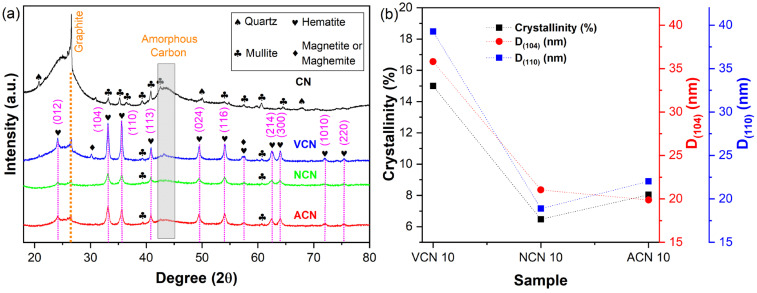
Structural analysis of samples subjected to thermal treatment under vacuum, nitrogen, and air atmospheres. (**a**) XRD patterns and (**b**) variation in hematite crystal size along the (104) and (110) directions, and estimated crystallinity of each nanocomposite.

The crystalline structure of the samples was analyzed using XRD. Both the annealed coal and the carbon-based nanocomposites coated with iron oxides were examined to identify the present phases. Figure 2 shows the diffractograms and structural parameters of the samples. In Figure 2a, the CN sample exhibits the characteristic peaks of graphite and amorphous carbon at 26.5°and approximately 42.3°, which is consistent with data reported in COD 9011577 [42]. Additional peaks of lower intensity are observed, corresponding to quartz (20.8°, 26. 5°, 36.4°, 40.2°, 42.3°, 50°, 68°, COD 9011493) [43] and mullite (25.9°, 26.1°, 30.9°, 33.1°, 35.1°, 36.9°, 39.1°, 40.8°, 42.5°, 60.5°, COD 9001321) [44]. Furthermore, it is important to note that the peaks widths around 26° and 42° are indicative of turbostratic carbon, a structure composed of both amorphous carbon and crystalline graphite phases, where each phase contributes carbon atoms in *sp*^3^ and *sp*^2^ hybridization states, respectively [45]. These results indicate that the carbon structure includes both amorphous carbon, evidenced by broad peaks, and crystalline graphite, along with silica and alumina.

In contrast, the sample thermally treated under vacuum (VCN 10) shows more intense peaks in the (104) and (110) directions, characteristic of the hematite phase (*α-*Fe_2_O_3_, peaks at 24.1°, 33.1°, 35.6°, 40.8°, 49.4°, 54.0°, 62.4°, 63.9°, and 71.9°, COD 2108027) [46], along with the amorphous and crystalline carbon phases. For the samples treated in nitrogen and air (NCN 10, ACN 10), a similar behavior was observed, although the intensities of the characteristic peaks vary. The NCN 10 sample shows the lowest intensity of hematite peaks, which can be attributed to the nitrogen environment, possibly limiting the crystallization of hematite. It should also be noted that thermal treatment may promote the formation of a second phase, such as magnetite (Fe_3_O_4_) and/or maghemite (γ-Fe_2_O_3_) in small amounts. However, due to the identical inverse spinel structure of these phases, they are difficult to distinguish by XRD analysis alone [47]. To complement this study, Mössbauer spectroscopy was conducted (vide infra).

Using Bragg’s law and Scherrer’s method [48,49,50,51,52], the crystal size and lattice parameters of hematite were calculated and reported in Table 1. No significant differences in lattice parameters were found across the different thermal treatments, suggesting that the crystal structure of hematite remains stable under the employed atmospheres.

Figure 2b presents the crystal size for hematite along the (104) and (110) directions (denoted as D_(104)_ and D_(110)_, respectively). The VCN 10 sample exhibits substantially larger crystal sizes compared to the NCN 10 and ACN 10 samples, indicating that both nitrogen and air atmospheres (the latter composed of ~78 wt.% nitrogen) constrains crystal growth. Given the amorphous nature of coal, the crystallinity of the CN-hematite nanocomposites, defined as the mass fraction of the crystalline regions within the material and representative of long-range structural order, was determined combining the reference intensity ratio (RIR) and degree of long-range (DOC) methods [53,54,55]. The RIR method relates crystallinity to amorphous phase content, while the DOC method quantifies crystalline phase directly. The crystallinity analysis revealed that the vacuum-treated nanocomposites reached the highest crystallinity (15%), which is nearly double that observed in the nitrogen- (6.5%) and air-treated (8.0%) samples. This trend mirrors the behavior observed for the crystal size (Figure 2b), suggesting that higher crystallinity facilitates the formation of larger hematite nanocrystals.

### 3.2. Electron Microscopy Analysis

**Figure 3:**
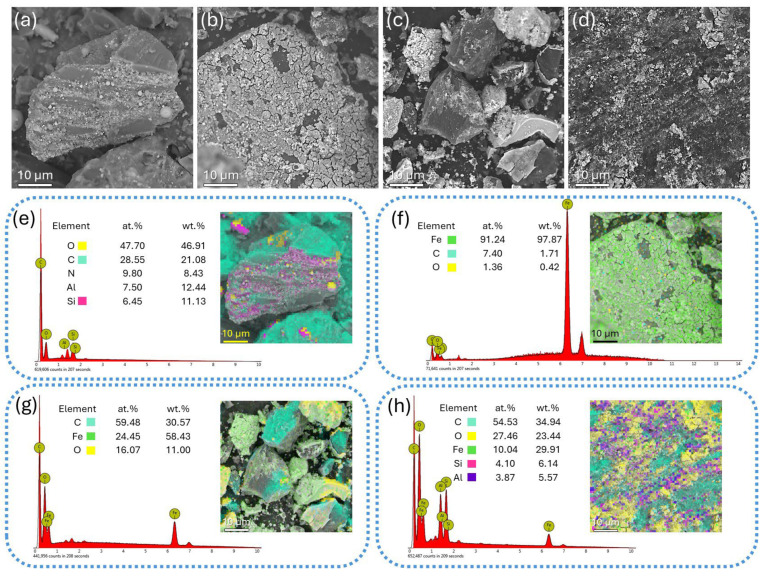
Morphology and elemental composition of the samples analyzed by SEM and EDS experiments. SEM images of (**a**) CN, (**b**) VCN 10, (**c**) NCN 10, and (**d**) ACN 10 samples. EDS compositional maps and elemental quantification of (**e**) CN, (**f**) VCN 10, (**g**) NCN 10, and (**h**) ACN 10 samples, highlighting the distribution of carbon, oxygen, iron, and trace elements.

Following the structural analysis, a microscopic examination by SEM and TEM experiments was carried out to evaluate the influence of different thermal treatment atmospheres on morphology and crystallinity of the nanocomposites. Figure 3 presents SEM images and EDS elemental mapping results, including atomic and weight percentage quantification, for both untreated coal powders (CN) and the synthesized nanocomposites. The SEM image of the CN sample (Figure 3a) shows micrometer-sized grains with irregular shapes and varying sizes. According to EDS analysis (Figure 3e), the coal grains extracted from the mine are primarily composed of carbon and oxygen, as expected, with trace amounts of nitrogen, aluminum, and silicon. The SEM image of the VCN 10 sample (Figure 3b) reveals that the iron-containing compound forms a coating on the surface of a carbon grain of tens of microns in size, as well as on adjacent smaller grains, as confirmed by the elemental map in Figure 3f. For the NCN 10 sample, the SEM image (Figure 3c) and EDS mapping (Figure 3g) show carbonaceous grains with generally smaller sizes compared to the CN and VCN 10 samples. Iron-based compounds are presents as surface deposits, though less homogeneously distributed. Finally, in the ACN 10 sample, the SEM image (Figure 3d) demonstrates that the thermal treatment in air results in a significant reduction in the size of the carbon-based grains, with dimensions ranging from a few microns down to hundreds of nanometers. EDS analysis (Figure 3h) indicates the presence of iron and oxygen on the carbon surface, but no evidence of a continuous coating over the entire grain was observed.

Thus, from the SEM-EDS analysis, it can be concluded that vacuum thermal treatment favors the effective deposition of iron oxide particles onto the carbon grain surface using the wet impregnation method. In contrast, thermal treatment in air promotes a notable reduction in the particle size of the carbon support.

**Figure 4:**
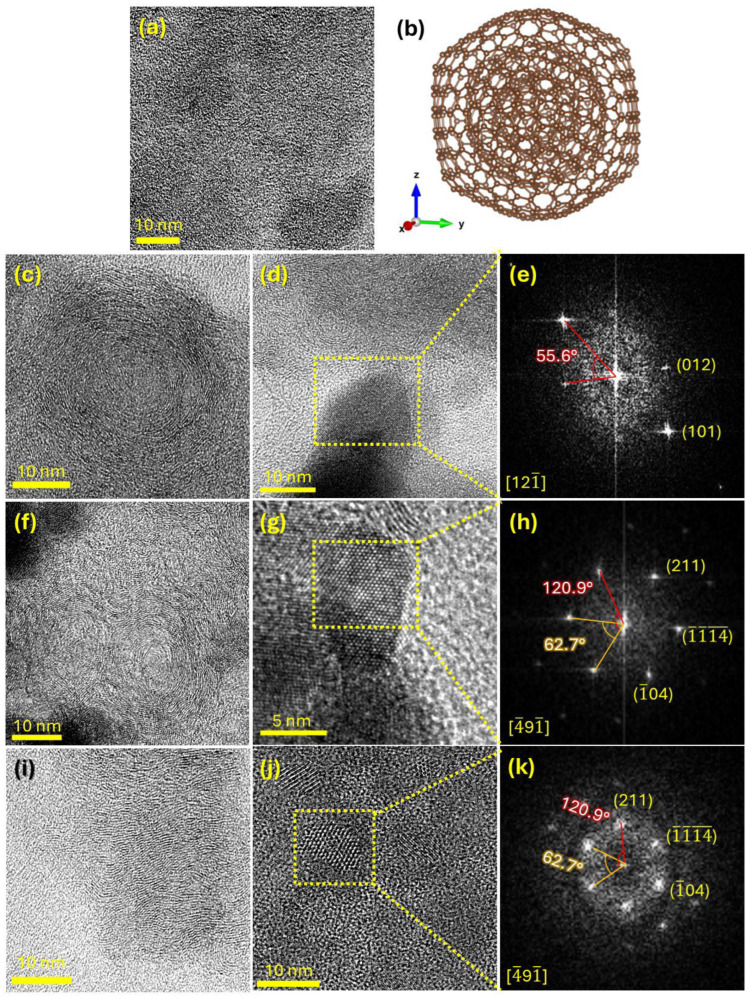
Morphological and structural analysis of the synthetized samples performed by TEM. HRTEM images acquired on carbon nanostructures of the (**a**) CN, (**c**) VCN 10, (**f**) NCN 10, and (**i**) ACN 10 samples. (**b**) Model of the nano-onion structure. HRTEM images recorded on hematite nanocrystals of the (**d**) VCN 10, (**g**) NCN 10, and (**j**) ACN 10 samples. (**e**,**h**,**k**) are FFT images reconstructed from the nanocrystals highlighted in the HRTEM images of (**d**), (**g**), and (**j**), respectively.

Figure 4 displays representative HRTEM images, along with a series of fast Fourier Transformed (FFT) images extracted from nanocrystals attached to the carbon nanostructures, in order to analyze the morphology of the nanocomposites at the nano- and atomic scale. Figure 4a shows an HRTEM image of a carbon nanostructure from the CN sample. Lattice fringes with very short-range order can be seen embedded within an amorphous matrix, indicating that the untreated coal is predominantly amorphous, consistent with the XRD results. In contrast, the HRTEM images of the thermally treated samples—VCN 10 (Figure 4c), NCN 10 (Figure 4f), and ACN 10 (Figure 4i)—demonstrate that all three thermal treatment conditions promote partial crystallization of the carbon phase. The VCN 10 sample reveals the formation of onion-like carbon nanostructures, similar to that schematically represented in Figure 4b [56,57]; the NCN 10 sample shows a mixture of curved-graphitic and onion-like carbon nanostructures; and the ACN 10 sample displays curved-graphitic carbon nanostructures. In all treated samples, small nanocrystals with sizes ranging from approximately 8 to 12 nm were identified in close proximity to the carbon nanostructures (Figure 4d,g,j). These nanocrystals were attributed to the hematite phase, based on electron diffraction analysis carried out on FFT images (see Figure 4e,h,k). These results confirm that the nanoscale morphology of the nanocomposites is consistent with the macroscopic structural trends observed in the XRD analysis.

### 3.3. Mössbauer and Raman Spectral Analysis

**Figure 5:**
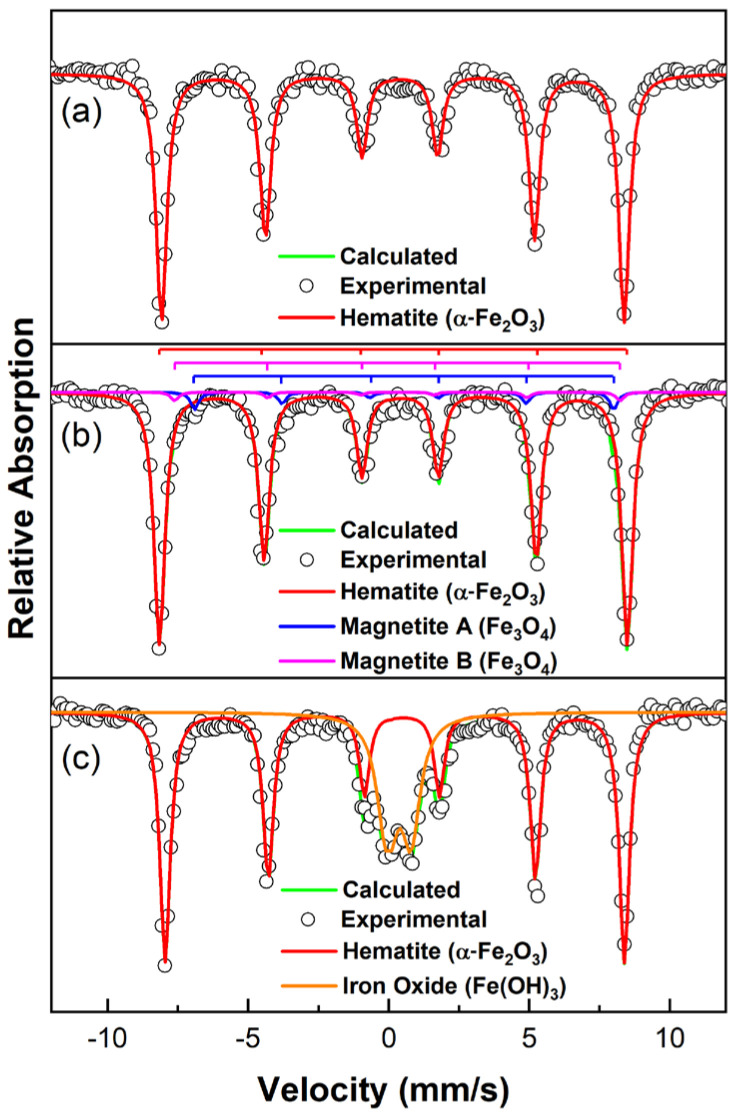
Mössbauer analysis for each sample. (**a**) ACN 10, (**b**) VCN 10, and (**c**) NCN 10. The experimental data is presented as open circles and the calculated fit in brown color line.

To identify the type of iron oxides present in each Fe_x_O_y_/C composite, room-temperature Mössbauer spectroscopy measurements were performed, as shown in Figure 5. Each spectrum exhibits well-defined magnetic hyperfine splitting, which can be accurately fitted using three sextets and one doublet. In the ACN 10 sample (Figure 5a), the spectral shape indicates that the composition consists of a single phase attributed to hematite (α-Fe_2_O_3_). This phase is specifically associated with Fe^3+^ ions occupying octahedral interstices formed by oxygen atoms, representing the most thermodynamically stable form of iron oxide [58,59]. For the VCN 10 sample (Figure 5b), two of the sextets correspond to the magnetite (Fe_3_O_4_) phase—one associated with Fe^3+^ ions in tetrahedral (A) sites and the other with Fe^2+^/Fe^3+^ ions in octahedral (B) site [60]. Additionally, another sextet is associated in greater proportion to hematite (α-Fe_2_O_3_) phase. In the NCN 10 sample (Figure 5c), the spectrum reveals a sextet corresponding to the hematite phase and a doublet attributed to amorphous iron hydroxide (Fe(OH)_3_). The Mössbauer parameters obtained from the spectral fitting are summarized in Table 2. The IS, QS, and B_HF_ values obtained for the fitted hematite phase are comparable with those reported in previous studies [61]. These values can be influenced by several factors, including the crystal size of iron oxide [62], the formation of layers leading to crystallization on the surface and within the carbonaceous material [58], and methodological aspects such as the sample preparation method and the thermal treatments to which the samples were subjected [59].

For the samples shown in Figure 5, the hyperfine parameters are slightly lower than those reported for ideal hematite. This discrepancy can be attributed to the possibility of non-homogeneous crystallization, leading to variations in particle size distribution [58]. Regarding the IS values, the results align with those typically observed for iron ions in high oxidation states and high-spin configurations [63], specifically Fe^3+^. Additionally, the negative QS values indicate a weak ferromagnetic character of hematite, which is consistent across all samples analyzed in this study, as expected for this phase [64,65].

As is observed in Figure 5a, the hematite phase obtained under an air atmosphere (ACN 10 sample) exhibited a relative area percentage of 100%, reinforcing the thermodynamic stability of this phase under oxidizing conditions. In contrast, for the magnetite (Fe_3_O_4_) phase in the VCN 10 sample (Figure 5b), the fitted relative areas suggest that site B occupies twice the area of site A, which is characteristic of stoichiometric magnetite. Notably, a secondary phase was detected only in the NCN 10 sample (Figure 5c), synthesized under an N_2_ atmosphere. This phase is likely associated with the formation of the Fe(OH)_3_.

**Scheme 1:**
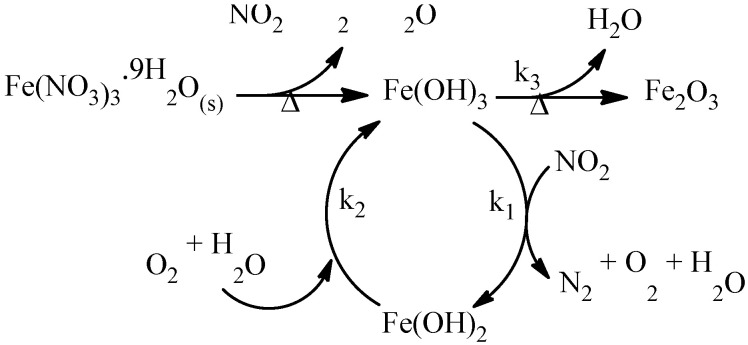
Thermal decomposition process of hydrated iron(III) nitrate nonahydrate in a static N_2_ atmosphere. Adapted from reference [66].

The Mössbauer parameters obtained in this study closely align with those reported by Euler and co-workers [66], who proposed the formation of this intermediate species during the decomposition of Fe(NO_3_)_3_⋅9H_2_O into Fe_2_O_3_. According to this mechanism, the decomposition process provides both reducing (NO_2_) and oxidizing (O_2_) agents, maintaining their concentrations relatively constant in a static N_2_ atmosphere. NO_2_ facilitates the formation of reduced iron in the Fe(OH)_2_ form, which subsequently re-oxidizes to Fe(OH)_3_ in the presence of oxygen and water molecules (see Scheme 1).

In our system, the absence of NO_2_, O_2_, and H_2_O, due to the use of a continuous N_2_ flow, prevents the reduction and re-oxidation processes between the Fe(OH)_3_ and Fe(OH)_2_ species. As a result, the transformation of Fe(OH)_3_ into Fe_2_O_3_ occurs through a kinetically favorable pathway. The presence of the Fe(OH)_3_ phase is evidenced by broad, low-intensity peaks in the XRD pattern, which are characteristic of its amorphous nature. In contrast, the presence of magnetite as a secondary phase in the VCN 10 sample (see Figure 5b) can be attributed to the conditions in a static vacuum atmosphere, where the formation of Fe(OH)_2_ is slightly favored due to the presence of reducing agents such as NO_2_ [66]. This adjustment was made to facilitate the accurate identification of other iron sites in the spectra.

**Figure 6:**
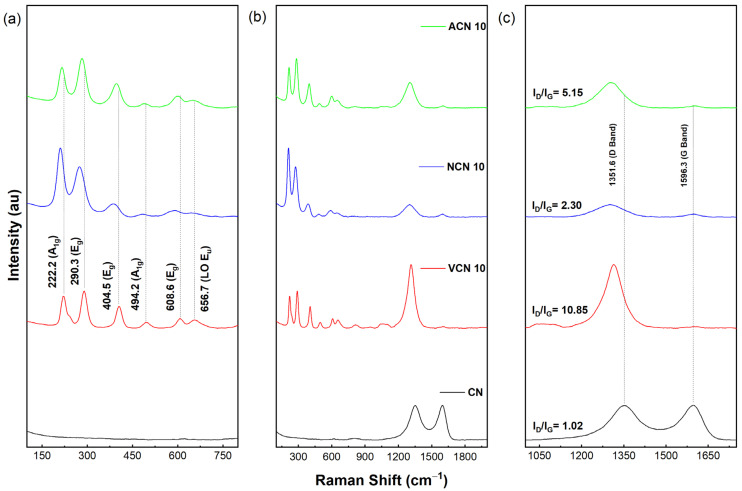
Raman spectra of all samples. Raman shift between (**a**) 150–750 cm^−1^, (**b**) 300–1800 cm^−1^, and (**c**) 1050–1650 cm^−1^.

The Raman spectra of all samples are shown in Figure 6. These spectra confirm that the majority phase corresponds to hematite; with six vibrational modes identified in the Raman region between 200 and 800 cm^−1^. These vibrational modes correspond to: A_1g_ phonon modes at approximately 222.2 cm^−1^ and 494.2 cm^−1^; E_g_ phonon modes at around 290.3 cm^−1^, 404.5 cm^−1^, and 608.6 cm^−1^; the relatively weak band around 656.7 cm^−1^ may arise from disorder-activated modes or longitudinal optical phonons consistent with partial structural disorder in hematite [67,68,69,70,71,72]. Our findings are consistent with previous reports, confirming the presence of these six vibrational modes. Additionally, the presence of a very weak vibrational band observed at around 656.7 cm^−1^ suggests structural disorder in the crystalline structure of hematite. No vibrational modes associated with Fe_3_O_4_ and Fe(OH)_3_ structures were observed, probably due to low concentration of these phases. Furthermore, two vibrational bands at around 1350 cm^−1^ and 1596 cm^−1^ (see Figure 6b,c) are attributed to carbonaceous materials. The D-band is observed to be prominent in all samples, while the G-band exhibits lower intensity, comparable to that of the D-band (see Figure 6c). The D-band is forbidden in a perfect graphitic material and becomes active only in the presence of disorder. The intensity ratio (*I_D_/I_G_*) is commonly used to indicate the degree of crystallinity in carbonaceous materials. Based on the *I_D_/I_G_* ratio, all samples exhibit a high degree of defects and amorphous carbon, which agrees with XRD and TEM analysis. Additionally, based on the synthesis conditions, a shift to lower wave numbers is observed for samples ACN 10 and NCN 10 compared to VCN 10 and CN. This red-shift may indicate a reduction in graphitic domain size, increased structural disorder, or changes in local strain induced by different atmospheric conditions during synthesis. Regarding ID/IG ratio, the highest value was observed for the VCN 10 sample, significantly exceeding those of the ACN 10 (5.15) and NCN 10 (2.30), and even the CN (1.02) sample. These results suggest that the vacuum thermal treatment induces the formation of nanocomposites with a higher degree of structural defects in the carbon phase.

### 3.4. Magnetic Analysis

**Figure 7:**
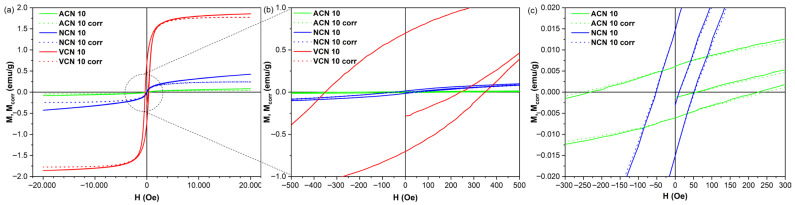
(**a**) Magnetization hysteresis loops of the synthesized composites. Zooming in (**b**,**c**) improves the visualization of the hysteretic behavior of the magnetization.

Figure 7 shows the magnetization (M) curves as a function of the applied magnetic field (H), obtained from room-temperature VSM experiments to investigate the magnetic behavior of the nanocomposites. All samples exhibit very narrow hysteresis loops, with a very low saturation magnetization (M_s_), and low remanent magnetization (M_r_) and coercive field (H_c_). This magnetic behavior is governed by the weak ferromagnetic nature of the hematite phase. At room temperature, hematite exhibits antiferromagnetic order with a small canted magnetic moment, which results in a weak ferromagnetic response [64,65]. The antiferromagnetic character of hematite is evidenced by the linear increase in magnetization in the reversible part of the hysteresis loop. For this reason, to estimate M_s_ of each nanocomposite, the hysteresis loops were corrected to subtract the antiferromagnetic contribution, considering that corrected magnetization (M_corr_) can be estimated as M_corr_ = M − NH, where N is the slope of the linear magnetization increase at high magnetic fields. Additionally, M_s_ normalized to the iron content (denoted here as M’_s_) was estimated for all samples, considering that the resulting carbon-based nanocomposites contain 9.77 ± 0.06 wt.% of Fe according to inductively coupled plasma mass spectroscopy (ICP-MS) results, which is very close to the expected value (10 wt.%). All the relevant magnetic parameters are listed in Table 3.

Regarding saturation magnetization, we found that both ACN 10 (virtually pure hematite) and NCN 10 (70.6 wt.% hematite) samples exhibited M’s values of 0.4 and 2.4 emu/g, respectively. These values fall within the same order of magnitude as those reported for hematite single crystals and nanoparticles (between 0.05 and 3 emu/g), a value that is highly sensible to different factors such as grain size, thermal treatment, and surface spin disorder [64,65]. In the case of NCN 10 sample, the Fe(OH)_3_ phase, which has an antiferromagnetic behavior at room temperature [73,74,75], contributed to an increased slope in the magnetization curve. The VCN sample showed the highest saturation magnetization (M’s = 17.7 emu/g), which can be attributed to the presence of magnetite nanoparticles in the nanocomposite, which present ferrimagnetic behavior with a saturation magnetization ranging from 41 to 80 emu/g [76]. It is important to note that all samples show non-negligible remanent magnetization and coercive field values, indicating the absence of superparamagnetic behavior. This can be attributed to the crystal size of the synthesized hematite phase, which exceeds the critical size threshold for superparamagnetism, estimated to be approximately 8–10 nm [77,78,79].

## 4. Conclusions

This study demonstrated that the thermal treatment atmosphere is a critical parameter influencing the structural and magnetic properties of iron oxides-coated carbon nanocomposites. Processing under vacuum conditions significantly enhanced crystallinity, with the vacuum-treated sample exhibiting approximately 15% hematite crystallinity, compared to ~8% under air and ~6.5% under nitrogen atmospheres. Furthermore, Mössbauer spectroscopy confirmed that the air-treated composite predominantly consisted of thermodynamically stable hematite, while the vacuum-treated composite contains a minor magnetite fraction, and the nitrogen-treated sample exhibited a mixed phase of hematite and amorphous iron hydroxide. These variations in phase composition and crystallinity directly impacted the magnetic behavior, with the vacuum-treated nanocomposites showing a well-defined ferromagnetic response, while the air- and nitrogen-treated samples exhibited only weak ferromagnetism.

Overall, these findings underscore the importance of tailoring the synthesis atmosphere to optimize the structural and magnetic properties of carbon-based iron oxide nanocomposites. By elucidating the influence of processing conditions in phase evolution and microstructural order, this work provides valuable insights for the rational design of advanced materials for applications such as environmental remediation, and energy conversion. The integrated approach presented herein paves the way for further exploration and development of high-performance nanocomposite systems, wherein precise control over the thermal treatment environment is paramount to achieving the targeted functional properties.

## Figures and Tables

**Table 1:** Structural parameters of hematite and crystallinity percentage of the nanocomposites.

Parameters	VCN 10	NCN 10	ACN 10
Hematite (α-Fe_2_O_3_)			
a (Å)	5.037 ± 0.005	5.039 ± 0.004	5.039 ± 0.004
c (Å)	13.74 ± 0.06	13.7 ± 0.1	13.7 ± 0.1
V (Å^3^)	302 ± 2	301 ± 4	301 ± 3
Crystal System	Rhombohedral		
Space Group	R-3 (148)	R-3 (148)	R-3 (148)
D_(104)_ (nm)	35.8 ± 0.4	21.0 ± 0.3	19.9 ± 0.2
D_(110)_ (nm)	39.3 ± 0.5	18.9 ± 0.2	22.0 ± 0.3
Nanocomposite			
Crystallinity (%)	15.0	6.5	8.0

**Table 2:** Hyperfine parameters obtained from the room-temperature Mössbauer spectra for all the Fe_x_O_y_/C composites: isomer shift (IS), quadrupole shift (QS), magnetic hyperfine field (B_HF_) at iron nuclei, and relative area (RA) of the components.

Sample	Phases	IS (mm/s)	QS (mm/s)	B_HF_ (kOe)	RA (%)
ACN 10	(α-Fe_2_O_3_) Fe^3+^	0.291	−0.251	508	100
VCN 10	(α-Fe_2_O_3_) Fe^3+^	0.307	−0.246	513	93
	(Fe_3_O_4_) Site A (Fe^3+^) (Fe_3_O_4_) Site B (Fe^2+^, Fe^3+^)	0.328	-	489	2.3
		0.568	-	460	4.6
NCN 10	(α-Fe_2_O_3_) Fe^3+^	0.429	−0.253	504	70.6
	(Fe(OH)_3_) Fe^3+^	0.439	0.784	-	29.3

**Table 3:** Magnetic parameters of the nanocomposites determined by VSM measurements.

Sample	M_r_ (emu/g) ±0.002	H_c_ (Oe)	M_s_ (emu/g) ±0.01	M’_s_ (emu/g) ±0.1
ACN 10	0.006	230.6 (6)	0.04	0.4
VCN 10	0.702	351 (2)	1.77	17.7
NCN 10	0.015	51.7 (1)	0.24	2.4

## Data Availability

The data presented in this study are available on request from the corresponding authors.
